# The Characterization of Columnar Apple Gene *MdCoL* Promoter and Its Response to Abscisic Acid, Brassinosteroid and Gibberellic Acid

**DOI:** 10.3390/ijms231810781

**Published:** 2022-09-15

**Authors:** Tingting Han, Jiahui Yu, Jie Zhuang, Ziyu Wang, Xin Sun, Yugang Zhang

**Affiliations:** 1 College of Horticulture, Qingdao Agricultural University, Qingdao 266109, China; 2 Engineering Laboratory of Genetic Improvement of Horticultural Crops of Shandong Province, Qingdao 266109, China

**Keywords:** ABA, BR, columnar apple, GA, *MdCoL* promoter

## Abstract

Columnar apple was an important germplasm resource to develop compact cultivars for labor-saving cultivation and to study fruit tree architecture. *MdCoL* is a strong candidate gene for controlling the columnar phenotype in apple. In this study, a 2000 bp upstream region of *MdCoL* was cloned as a full-length promoter, named *MdCoLp1*. To gain a better understanding of the characterization of the *MdCoL* promoter, cis-acting elements and the binding sites of transcription factors were predicted and analyzed, and four binary expression vectors consisting of the GUS reporter gene under the control of the *MdCoL* promoter was transformed into *Arabidopsis thaliana* to analyze the response to abscisic acid (ABA), brassinosteroid (BR) and gibberellic acid (GA_3_) of *MdCoL* promoters. Multiple transcription factors involving TCP, BEL1 and BES1/BZR1 and other transcription factor (TF) binding sites were predicted on the promoter of *MdCoL*. Histochemical staining showed that both full-length and 5′ truncated promoters could initiate GUS expression. The GUS activity was the most in leaf and stem, and mainly concentrated in the fibrovascular tissue, followed by root, and the least activity was observed in silique and flower. In addition, *MdCoL* expression was mainly localized in the quiescent center (QC) and lateral root growing point of root tip and the vascular tissue of stem and leaf by in situ hybridization. The results of exogenous hormones treatment showed that ABA and BR could activate the activity of the *MdCoL* promoter, while GA_3_ had opposite effects. In columnar apple seedlings, ABA treatment could upregulate the expression of *MdCoL*, but GA_3_ and BR restrained the transcription level of *MdCoL*. These results provide the foundation for deciphering the regulatory network of hormones affecting *MdCoL* transcription.

## 1. Introduction

Plant architecture is a major determinant of photosynthesis, resource allocation, cultivation pattern and crop productivity [1]. Columnar apple tree exhibits short internodes, limited side branches and increased spurs, which were initially identified in the 1960s as a sort of normal structure varieties of ‘McIntosh’ (Mc) named ‘McIntosh Wijcik’ (Wi) [2]. The columnar type was considered as an ideal architecture that could improve photosynthetic utilization and facilitate mechanized management. Classical genetic studies proved that the columnar growth habit is a quality trait controlled by a single dominant gene *Co* [3,4,5,6]. Genetic linkage maps had been constructed and DNA molecular markers had been developed for identifying the *Co* gene and the columnar phenotype. Finally, the *Co* gene was located on linkage group 10 [4,5,7,8,9,10]. Later studies have found that there is a DNA element/Gypsy retrotransposon that was inserted in the *Co* region of columnar apples, and the expression of *MdCoL*/*MdCo31*/91071-gene located 16kb downstream of insertion in columnar apples is significantly upregulated compared with standard apples [10,11,12,13]. There were other candidate genes in or around the *Co* region; RT-PCR analysis showed that *MdCoL* was expressed in the buds, shoot apexes and leaves of ‘McIntosh Wijcik’ in aseptic culture [10,11,12,13]. Furthermore, *Arabidopsis* and tobacco overexpressed *MdCoL* and are more dwarfed than their wild type, and both have short internodes which are similar to columnar apples [10,12]. Therefore, *MdCoL* was considered a strong candidate gene for controlling the columnar phenotype in apple. 

Phytohormones play a principal role in regulating plant architecture. ABA was well-established as a stress-response hormone. Recently, some studies showed that ABA also regulates plant branching. The strigolactones (SL) signaling pathway could upregulate the expression of *OsHOX12* by increasing the expression level of *OsNCED1*, a key gene for ABA synthesis, and then promoting the synthesis of ABA in shoot base, thereby inhibiting the elongation of lateral buds [3]. *BRC1*, specifically expressed in lateral buds, could promote the expression of ABA biosynthetic genes to increase the content of ABA in buds, thus causing inhibition of bud development [14,15,16]. GA has provided multiple biological functions, the most important of which is to promote the elongation of internodes [17]. An SNP mutation occurred in GA receptor GID1, resulting in a GA insensitive dwarf type in peach [18]. BR is a group of steroid phytohormones that also plays an important role in regulating plant architecture [19,20]. Several reports showed that mutations in BR synthesis and signaling genes result in lower tiller number [21,22]. BR directly repressed the transcription of *BRC1* though the key transcription factor BZR1 and relieved its inhibitory effect on lateral bud activation [23]. In addition to this, some studies have shown that there are complex regulatory relationships among BR, GA and ABA. BR could promote GA accumulation by regulating the expression of GA metabolic genes *GA3ox-2* [22]. ABA could induce the expression of *OsGSR1*, a positive regulator of GA signaling, and *OsGSR1* could activate BR synthesis by regulating a BR biosynthetic enzyme at the post-translational level directly [24,25]. MdBZR1, a transcription factor of the BR signal transduction pathway, directly binds to the promoter of *MdABI5* and suppresses its expression to mediate ABA response [26]. 

*MdCoL* encodes a putative 2OG-Fe(II) oxygenase or downy mildew resistant 6 (DMR6) [10,11]. The homologous gene in *Arabidopsis thaliana*, *AtDMR6* (AT5G245301.1), also encodes a putative 2OG-Fe(II) oxygenase that is defense-associated but required for susceptibility to downy mildew [27]. Studies have shown that *dmr6* was resistant to the bacterium Pseudomonas syringae and the *Oomycete Phytophthora capsici* and has a cross-talk between the flavone and salicylic acid (SA) pathways in Arabidopsis [28,29]. Further studies revealed that DMR6 could act as an SA 5-hydroxylase (S5H) that catalyzes the formation of 2,5-DHBA by hydroxylating SA at the C5 position of its phenyl ring [30]. At the same time, *MdCoL* was also confirmed to restrain the synthesis of bioactive GAs via leading to 12-hydroxylation of GA_12_ in the process of GAs synthesis while the formation of columnar characteristics due to GA deficiency in columnar apple occurred [13,14,15,16]. Our previous studies had shown that *MdCoL* could improve the biosynthesis of ABA via upregulating the expression of *MdNCED6/9*, which may be one of the reasons for inhibiting the branch growth of columnar apple [13]. Multiple hormones regulated the columnar phenotypes in apple. Although the *MdCoL* could affect hormone levels in columnar apple, it was unclear whether it was also regulated by a variety of hormones.

In the current study, we report the characterization of the columnar gene *MdCoL* promoter in apple firstly and revealed that GA and ABA could regulate the expression of *MdCoL* in a feedback regulatory loop to further affect the formation of columnar traits. These efforts will provide significant information for the analysis of *MdCoL* function and regulatory network.

## 2. Results

### 2.1. Isolation and Bioinformatics Analysis of MdCoL Promoter

**Figure 1:**
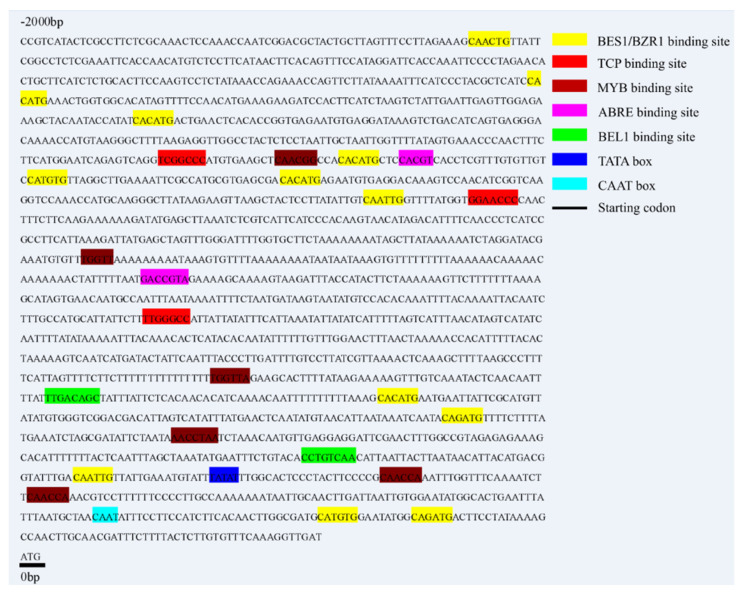
The nucleotide sequence and bioinformatics analysis of *MdCoL* promoter by PlantCARE and PlantRegMap. Different colored blocks represent different transcription factors’ binding sites or cis-acting elements. The short black line shows the start codon of *MdCoL*.

To identify the promoter sequence for regulatory motifs, the sequence of 2000 bp upstream of the *MdCoL* gene was cloned and submitted to the PlantCARE database and PlantRegMap for bioinformatics analysis. As shown in Figure 1, some cis-regulatory elements and TF binding sites were mapped in the *MdCoL* promoter. TATA-box and CAAT-box were 252 bp and 113 bp upstream of the start codon. In addition, we identified two ABA response elements ABRE (−1481 bp, −1054 bp), six MYB binding sites (−201 bp, −226 bp, −415 bp, −646 bp, −1139 bp, −1499 bp), three TCP binding sites (−897 bp, −1316 bp, −1516 bp), two BEL1 binding sites (−318 bp, −593 bp), and twelve BES1/BZR1 binding sites (−65 bp, −79 bp, −273 bp, −454 bp, −543 bp, −1332 bp, −1421 bp, −1458 bp, −1490 bp, −1510 bp, −1673 bp, −1769 bp, −1934 bp).

### 2.2. The Tissue Expression Characteristics of MdCoL in Columnar Apple

**Figure 2:**
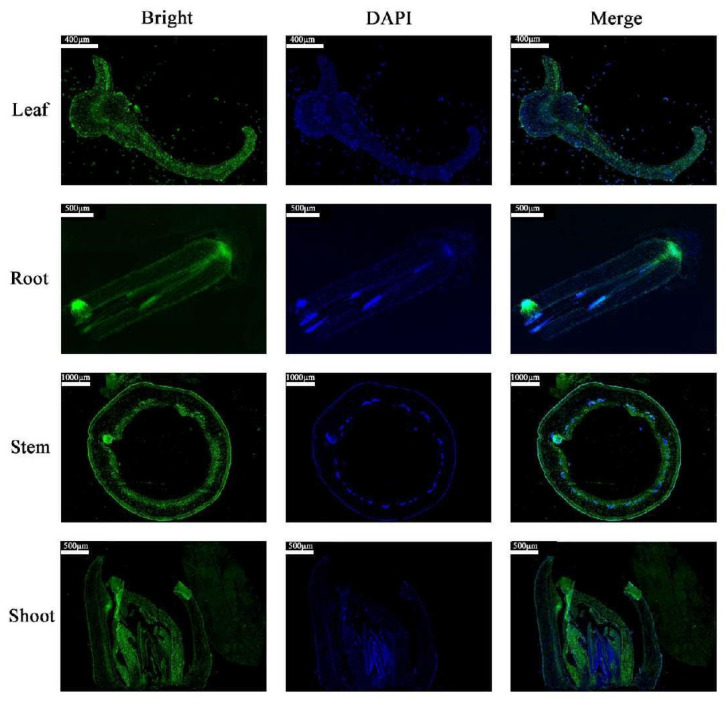
In situ hybridization of mRNA of *MdCoL* in leaf, root, stem and bud of columnar apple ‘Wijcik McIntosh’. “Bright” means the fluorescent signals were observed directly, “DAPI” means the fluorescence signal after DAPI (2-(4-Amidinophenyl)-6-indolecarbamidine dihydrochloride) staining. “Merge” represents the superposition image of the above two fluorescence signals.

The expression of *MdCoL* was examined in various tissues of columnar apple Wi and standard apple Mc through qRT-PCR in our group in 2021; the stronger expression of *MdCoL* was observed in leaves, roots, shoot tips (stems) and buds of Wi, with the highest expression was in stems [13]. In order to facilitate the tissue positioning characteristics of *MdCoL* in columnar apple, and thus to analyze the relationship between the target gene and the columnar habits of apple more clearly, we performed in situ hybridization in leaves, roots, stems and buds of Wi in this study. The immunolocalization showed that the transcripts of *MdCoL* were accumulated in stems, leaves, buds and root tips, but the signal mainly reflected in the QC and lateral root growing point of root tips, spongy and palisade tissue of leaves, and vascular tissue of shoots and leaves (Figure 2).

### 2.3. GUS Staining in Different Transgenic Arabidopsis Plants

**Figure 3:**
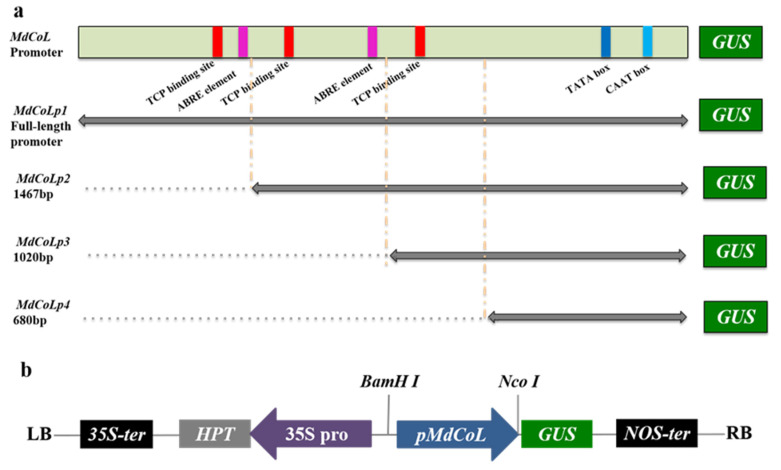
The scheme of *MdCoL* promoter constructs for Arabidopsis transformation. (**a**) The constructs diagram of *MdCoL* promoter with different length. (**b**) Schematic diagram of the expression vector for promoter activity detection, in which the expression vector pCambia1301 was used to clone the *MdCoL* promoter at the BamHI and NcoI sites. The transcription factors’ binding sites and cis-acting elements located on the *MdCoL* promoter were indicated by squares of different colors.

**Figure 4:**
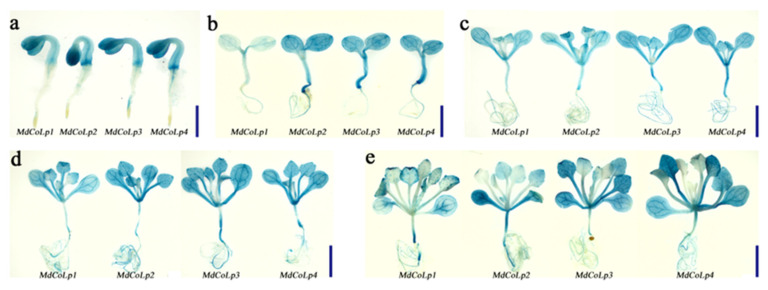
Histochemical staining of GUS in Arabidopsis plants transformed with the MdCoLp1/2/3/4::GUS construct. The age of Arabidopsis seedlings in (**a**–**e**) were 2 days, 5 days, 12 days, 18 days and 25 days, respectively. The scale bar of (**a**–**e**) is 2 mm.

**Figure 5:**
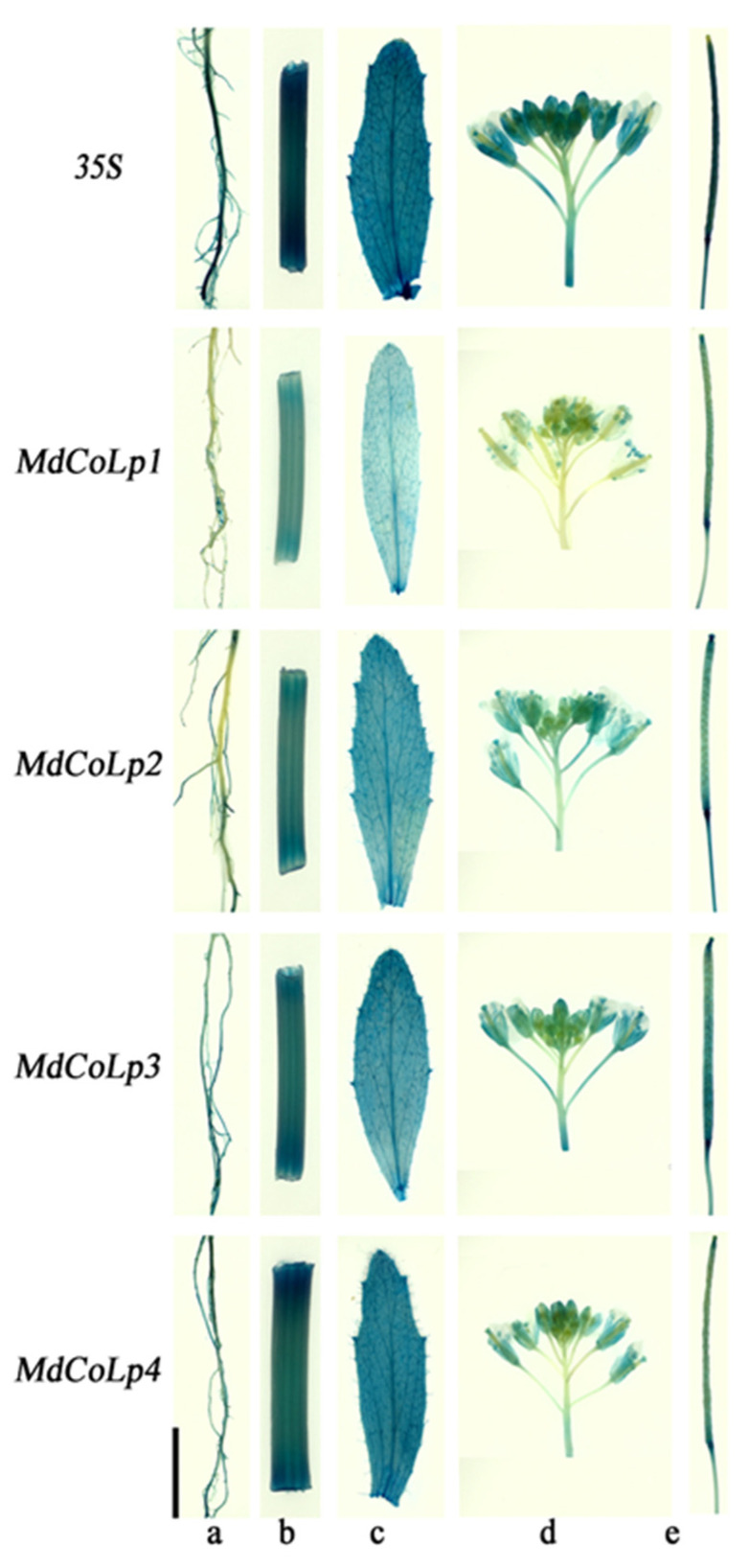
Histochemical staining of GUS in roots (**a**), stems (**b**), cauline leaves (**c**), inflorescences (**d**) and siliques (**e**) of Arabidopsis transformed with the construct of MdCoLp1/2/3/4::GUS and 35S::GUS at 35 days of seedlings Scale bar is 2 mm.

To determine whether the full-length *MdCoL* promoter and its 5’ truncated versions had transcriptional activity in Arabidopsis, GUS-reporter vectors containing different *MdCoL* promoter fragments or 35S promoter were prepared to transform Arabidopsis, obtaining five groups of transgenic Arabidopsis (Figure 3, Supplementary Figure S1). A variety of transgenic Arabidopsis organs (siliques, stems, leaves and flowers) with different truncated versions were stained by GUS staining. The staining assays in Arabidopsis showed that all truncated versions in transgenic lines exhibited stronger GUS signals than full-length promoters (Figure 4 and Figure 5), especially in Figure 5. Therefore, it is speculated that there is a sequence for an active repressor between the full-length promoter *MdCoLp1* and the 5’ truncated promoter *MdCoLp2*. Furthermore, leaves and stems had stronger GUS immunostaining than the other three organs (Figure 4 and Figure 5). It can be also seen that the activity level of GUS signals induced by *MdCoLp1* were the lowest in all truncated versions’ transgenic lines (Figure 4 and Figure 5). The highest GUS activity was observed in the stems of MdCoLp2 and MdCoLp3 (Figure 5). According to the results mentioned above, the GUS staining in transgenic Arabidopsis with different *MdCoL* promoter fragments was observed, and the GUS activity was the most in leaves and stems, in which, GUS staining was mainly concentrated in the fibrovascular tissue, followed by roots, and the least activity was observed in siliques and flowers. The activity of GUS in leaves was strong and stable, so we chose leaves as materials in the next hormones treatment test.

### 2.4. GUS Activity in Different Transgenic Arabidopsis Plants under Hormones Treatment

**Figure 6:**
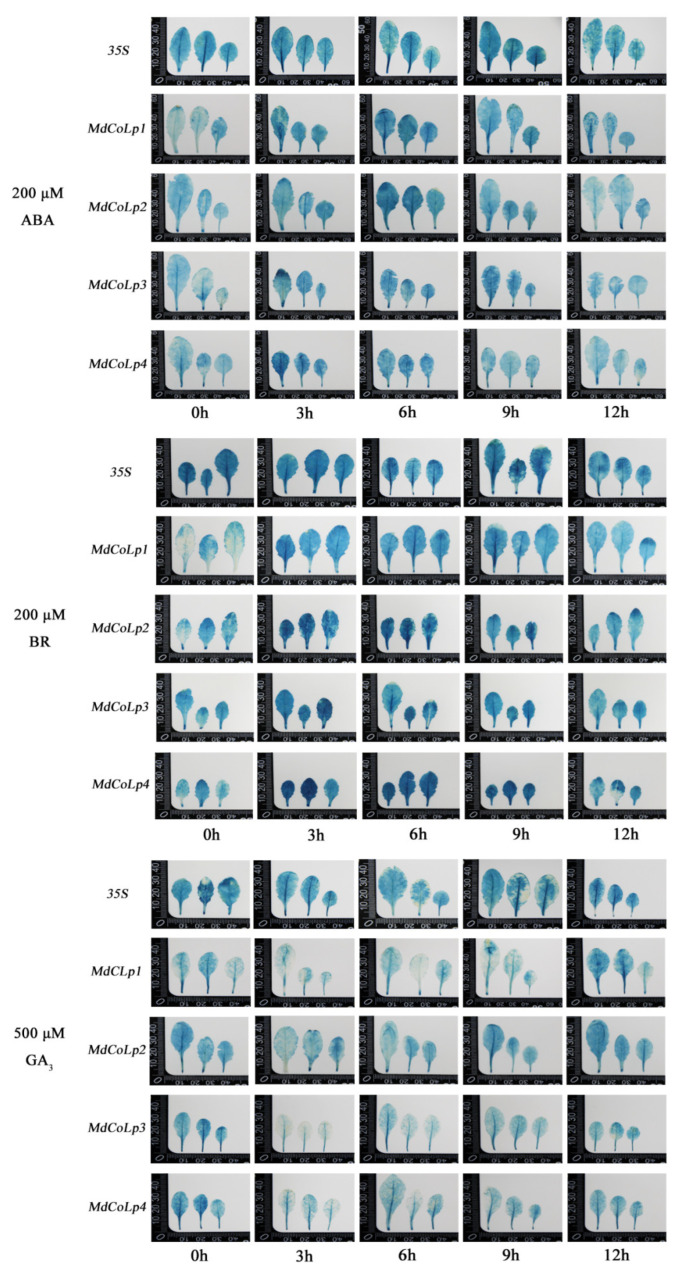
Effect of exogenous hormones treatment on the GUS histochemical staining of transgenic Arabidopsis plants transformed with the construct of MdCoLp1/2/3/4::GUS and 35S::GUS. The leaves used for 200 μM ABA, 200 μM BR and 500 μM GA_3_ treatment were rosette leaves of transgenic Arabidopsis growing normally for 30 days. A set of three leaves represent three transgenic Arabidopsis lines, respectively.

**Figure 7:**
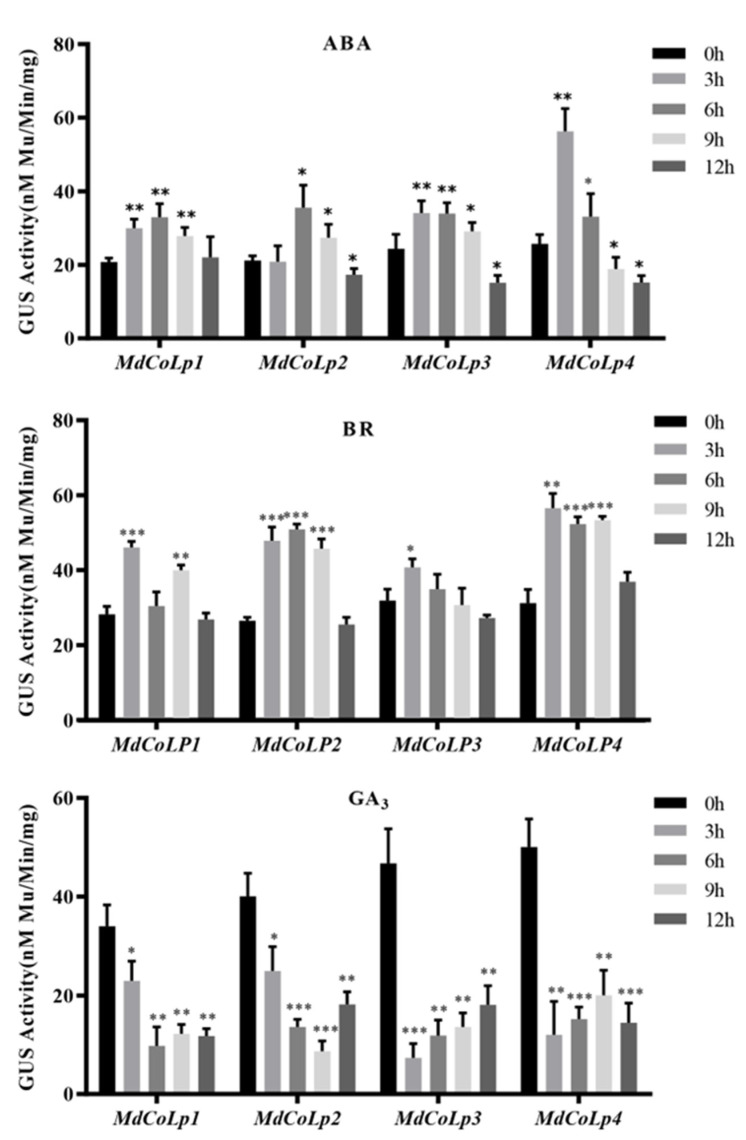
The effects of different hormones treatments on GUS activity under the control of different *MdCoL* promoter deletion segments in transgenic Arabidopsis lines. The GUS activity was detected in transgenic lines at 0, 3, 6, 9 and 12 h after treatment with 200 μM ABA, 200 μM BR and 500 μM GA_3_. Error bars indicate standard deviation from three independent biological replicates (* *p* < 0.05; ** *p* < 0.01; *** *p* < 0.001).

The plant architecture, especially apple columnar traits, was closely related to plant hormones. A series of hormone-related cis-regulatory elements and TF binding sites were predicted on the *MdCoL* promoter (Figure 1) to further explore the relationship between plant hormones and columnar traits and clarify the response of *MdCoL* to plant hormones. Five groups of transgenic Arabidopsis with different promoters were stimulated by ABA (200 μM), GA_3_ (500 μM), BR (200 μM) and staining by GUS staining solution (Figure 6). Four groups of transgenic Arabidopsis with different lengths of *MdCoL* promoters in them were collected to measure GUS activity after ABA, GA_3_ and BR treated (Figure 7). Our results show that ABA and BR treatment significantly enhanced the GUS activity of all transgenic lines, whereas the GUS activity after GA_3_ treatment was inhibited (Figure 6 and Figure 7).

GUS staining results of MdCoLp0, the positive control plants, fused with 35S promoter were stable after treat with ABA for 1–12 h (Figure 6). MdCoLp1 and MdCoLp2 showed the deepest GUS staining at 6 h of ABA treatment, while MdCoLp4 had the highest increase at 3 h of ABA treatment (Figure 6). There was no significant difference in GUS staining of MdCoLp3 from 3 to 9 h after ABA treatment, and both of them were deeper than 0 h (Figure 6). The results of GUS activity showed that MdCoLp1, MdCoLp3 and MdCoLp4 were significantly enhanced at 3–9 h compared with 0 h, and MdCoLp2 was significantly enhanced at 6–9 h (Figure 7). After 12 h of ABA treatment, GUS activity in four transgenic lines presented downregulation to varying degrees (Figure 7). The changes of GUS activity in all lines were consistent with the results of GUS histochemical staining.

BR and ABA treatments were similar in four promoter deletions’ transgenic lines, all of which could increase the expression of GUS (Figure 6 and Figure 7). The GUS staining of MdCoLp0 did not change after BR treatment, and the staining levels of the other four Arabidopsis groups were deepened after BR treatment (Figure 6). The GUS activity of MdCoLp1 treated with BR for 3 h and 9 h was significantly higher than that of control at 0 h (Figure 7). In MdCoLp2 and MdCoLp4, the GUS activity increased after 3 h of BR treatment and recovered after 12 h of BR treatment (Figure 7). The trend of change in MdCoLp3 started rising after 3 h until it began to recover after 9 h, and its GUS activity was less upregulated after BR treatment: only after 3 h of treatment were significant differences from untreated controls shown (Figure 7). 

Unlike ABA and BR treatment, GA_3_ treatment could reduce GUS expression in different 5’ truncated versions’ transgenic lines (Figure 6 and Figure 7). GUS staining results of MdCoLp0 were stable after treatment with GA_3_ (Figure 6). In MdCoLp1, the GUS staining degree after GA_3_ treatment was weakened and gradually disappeared compared with 0 h (Figure 6). The GUS staining of MdCoLp2, MdCoLp3 and MdCoLp4 decreased first and then recovered gradually with the extension of treatment time (Figure 6). The GUS activity of MdCoLp1 and MdCoLp2 also decreased first and then increased after GA_3_ treatment and reached the minimum value after GA_3_ treatment for 6 h and 9 h, respectively (Figure 7). The GUS activity in MdCoLp3 and MdCoLp4 decreased significantly after 3 h of GA_3_ treatment and resumed after 6 h (Figure 7). These results were consistent with the results of GUS staining.

### 2.5. MdCoL Promoter Affected MdCoL Expression under Hormones Treatment

**Figure 8:**
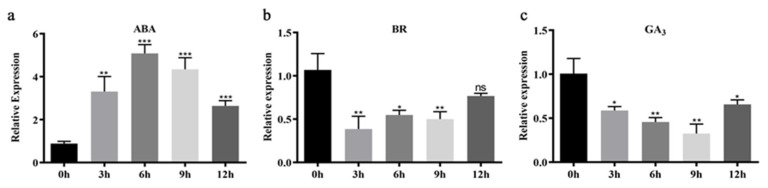
The expression of *MdCoL* in columnar apple ‘Wijcik McIntosh’ under different hormones treatments. The expression levels were detected in ‘Wijcik McIntosh’ seedlings at 0, 3, 6, 9 and 12 h after treatment with 200 μM ABA (**a**), 200 μM BR (**b**) and 500 μM GA_3_ (**c**). Error bars show standard deviation from three independent biological replicates (* *p* < 0.05; ** *p* < 0.01; *** *p* < 0.001; “ns” means no significant difference).

Consistent with the above results, the columnar apple seedlings treated with ABA, BR and GA_3_ showed time-dependent changes in *MdCoL* expression (Figure 8). After 3 h of ABA treatment, the levels of *MdCoL* expression increased 3.2 times compared to that of the baseline, slowly increased to the peak, and was followed by a downward trend after 9 h (Figure 8). The change of *MdCoL* expression in columnar apple seedlings after BR treatment was contrary to GUS activity analysis results of transgenic Arabidopsis. After 3 h with BR treatment, the expression of *MdCoL* was significantly decreased, and the subsequent treatment period remained basically unchanged (Figure 8). On the other hand, GA_3_ could downregulate *MdCoL* levels relative to the baseline after 3 h significantly; then, they gradually decreased and showed an upward trend after 12 h (Figure 8).

## 3. Discussions

Since its discovery in the last century, columnar apple has been an important germplasm resource for scientists to study the structure of apple trees and breeding varieties which are suitable for high-density, low-cost and labor-saving cultivation. Although breeders have developed many new varieties by combining columnar habits with other advantageous characteristics through traditional crossbreeding, the formation mechanism of the columnar phenotype is still unclear. Previous studies shown that *MdCoL* is a potential candidate gene involved in this process, and it was accumulated higher in aerial parts of columnar than in standard apple [10,11,12,13]. In this study, in situ hybridization results showed that the expression of *MdCoL* was abundant in leaves, stems, shoots and roots of Wi (Figure 3). Similar results were also observed in the GUS staining of transgenic Arabidopsis with different promoter fragments (Figure 5). These results could further prove that *MdCoL* is related to the formation of columnar traits, including short internodes, poor lateral branch formation, increased spurs, thick green leaves and a well-developed root system. 

Some studies had shown that the significant upregulation expression of *MdCoL* in aerial parts of columnar apple was due to the insertion of a retrotransposon upstream of the promoter [10,11,12]. Interestingly, previous studies, including ours and other groups, found that the transcription of *MdCoL* also accumulated highly in the roots of standard apple [13,31]. The *MdCoL* promoter was active in all tissues of transgenic Arabidopsis, and the above results indicated that there were other regulatory elements that affect the expression of *MdCoL*. In the current study, ABA, BR and GA_3_ could affect the activity of *MdCoL* promoter with different length in transgenic Arabidopsis, then regulate transcription levels of *MdCoL* (Figure 6, Figure 7 and Figure 8). The analysis of promoter showed that *MdCoL* promoter contained the binding sites of TCP, BEL1 and BES1/BZR1, and some TFs are relevant to phytohormone (Figure 1). Therefore, it is speculated that the transcription of *MdCoL* should be regulated by a series of TFs. Some studies have shown that genes of these transcription factor families may be involved in the formation of plant architecture by affecting hormone synthesis and signal transduction [15,23,32,33]. As the branching regulators, the TCP transcription factor TB1 could restrict the elongation of stem internodes and also induce bud suppression through increasing the production of ABA and JA and reducing sugar levels and energy balance [34,35]. The cucumber TCP family gene *CsBRC1* could reduce the accumulation of auxin in buds by directly repressing the expression of *CsPIN3*, a transporter of auxin, resulting in inhibition of the lateral bud outgrowth [36]. BZR1, the BR signaling component, could bind to *BCR1* directly, regulating its transcription and then promoting the growth of tomato buds through integrating GA, CK, SL and other hormonal pathways by it [23]. Thus, the transcription factor binding site on the *MdCoL* promoter region is likely to regulate the formation of apple columnar traits by recruiting specific TFs in response to plant hormones. The prediction of cis-acting elements and the transcription factor binding site of the *MdCoL* promoter is helpful to improve the regulatory mechanism and network of *MdCoL* in the formation of columnar traits.

The GUS activity of the full-length promoter was the weakest among the four promoters mentioned above; compared with the full-length promoter *MdCoLp1* and the three short promoters *MdCoLp2*, *MdCoLp3* and *MdCoLp4*, there was an extra 533 bp promoter sequence in *MdCoLp1*. Except for ABRE and the TCP binding site, this region may contain a binding site for an inhibitor that reduces the activity of *MdCoLp1*. 

Phytohormones play a vital role in the formation and regulation of plant architecture, including columnar habits in apple [6,13,37,38]. GA and ABA were key factors in regulating plant architecture [16,18,39]. The *MdCoL* could lead the formation of columnar traits through decreasing the biosynthesis of biologically active GAs and improving the biosynthesis of ABA, resulting in internode shortening and lateral branch growth in a slow manner [13,37,40]. The expression of GUS driven by those promoters, or the degree of GUS staining, showed a downward trend after GA treatment, and an upward trend after ABA treatment, although there were some differences in details. In other words, ABA could upregulate the activity of the *MdCoL* promoter with different lengths, and GA_3_ could downregulate the activity of the *MdCoL* promoter (Figure 6 and Figure 7). The results of qRT-PCR show that ABA upregulated the expression of *MdCoL*, and GA_3_ downregulated the expression of *MdCoL*, which also confirmed the above conclusion (Figure 8). On the whole, *MdCoL* did not only influence the formation of apple columnar traits by regulating the biosynthesis of ABA and GA, but also was upregulated by ABA and downregulated by GA_3_ in a feedback regulatory loop. 

Promoters do not regulate gene expression themselves but play a role by binding to TFs. There are many cis-elements and TF binding sites on promoters, which could help to predict their expression characteristics and functions. ABRE was predicted at two locations (−519 bp, 946 bp) in *MdCoL* promoters (*MdCoLp1* and *MdCoLp2*) (Figure 1 and Figure 3). Therefore, the presence of ABRE might be caused by the strong response of *MdCoLp1* and *MdCoLp2* to ABA treatment. Some MYB genes’ expression was induced by ABA but was repressed by GA, and these genes regulated cell-wall biosynthesis and phytohormone signaling [41,42]. Although the ABRE elements were absent at *MdCoLp3* and *MdCoLp4*, they had multiple MYB binding sites (Figure 1 and Figure 3); this activity could also be induced by ABA. The inhibition of GUS activity by GA_3_ in four promoter fragments as well as *MdCoL* expression may also be due to the presence of MYB sites. CANNTG were cis-elements associated with BR and were binding sites of BZR1 [38,43], though they were predicted in four promoter fragments. Therefore, in transgenic Arabidopsis, BR could promote the activity of promoters (Figure 6 and Figure 7). Interestingly, the expression of *MdCoL* was decreased after treatment of BR on the columnar apple ‘Wijcik McIntosh’ seedlings, and the expression of *MdCoL* was decreased (Figure 8). This may be due to the high content of ABA and low content of GA in columnar apple and the presence of coregulation of other genes. 

In this study, the promoter of columnar gene *MdCoL* was cloned, and a series of analyses and evaluations was conducted. The *MdCoL* promoter was able to respond to ABA, BR and GA_3_, and these hormones could regulate the expression of *MdCoL* (Figure 6, Figure 7 and Figure 8). A lack of active GAs and high levels of ABA could lead to the emergence of columnar traits. Moreover, GA_3_ and ABA could regulate the expression of *MdCoL* in a feedback loop to affect the formation of columnar traits. Further studies are in progress to explain the detailed mechanism of different hormones, especially BR, which regulated the formation of columnar type by influencing the expression of *MdCoL*.

## 4. Materials and Methods

### 4.1. Plant Materials and Treatments 

The apple materials were obtained from tissue cultured columnar apple Wi. The wild type Columbia *Arabidopsis thaliana* (Col-0) was used for genetic transformation in this study. Transgenic Arabidopsis and Wi seedlings were treated with ABA (200 μM), GA_3_ (500 μM) and BR (200 μM). Then, rosette leaves of transgenic Arabidopsis were sampled at 0, 3, 6, 9 and 12 h, and were used for histochemical GUS staining and GUS activity assays. In addition, *MdCoL* gene expression was performed in leaves of Wi seedlings at 0, 3, 6, 9 and 12 h through qRT-PCR after treatment. 

### 4.2. In Situ Hybridization

The *MdCoL*-specific probe labelled with FAM (carboxyfluorescein) was prepared from the unique region of coding sequence, and its nucleotide sequence was 5’-TGGGCATGGCGGATAATGGTTGGACAG-3’. Apical tips, leaves, stems and buds approximately 5.0 mm in length were collected from the tissue cultured seedling of columnar apple Wi, which were grown approximately one month in rooting medium after subculturing for about one month and fixed with RNase-free FAA solution at 4 °C overnight, respectively. Tissue sections and RNA hybridization were performed according to previous experimental methods with minor modifications [44]. Paraffin blocks were trimmed to make slices 10 μm thick; among them, stems and leaves were cut crosswise, and root tips and buds were cut longitudinally. The hybridization was carried out at 55 °C for 16 h, then observed and photographed by CaseViewer.

### 4.3. Isolation and Bioinformatics Analysis of MdCoL Promoter 

The genomic DNA was extracted from columnar apple Wi with TIANGEN Plant Genomic DNA Kit (TIANGEN BIOTECH, Beijing, China). The 2000 bp sequence upstream of the start codon of *MdCoL* was used as the promoter region [45]. Both the full-length and three 5’ truncated promoter fragments were isolated by PCR amplification using specific primers MdCoLp1-F, MdCoLp2-F, MdCoLp3-F, MdCoLp4-F and MdCoLp1-R (Supplementary Table S1). PCR products were cloned into the pMD-19T-vector and sequenced. The full-length promoter was submitted to PlantRegMap (http://plantregmap.gao-lab.org/binding_site_prediction.php accessed on 5 March 2020) to predict the binding site of transcription factors [46]. The online tool PlantCARE (http://bioinformatics.psb.ugent.be/webtools/plantcare/html/ accessed on 5 March 2020) was used to predict cis-regulatory elements.

### 4.4. Construction of GUS Reporter Expression Vectors and Genetic Transformation of Arabidopsis 

The full-length *MdCoL* promoter and three 5’ truncated promoter fragments were ligated into *pCAMBIA1301*, a binary expression vector with GUS reporter gene, to replace CaMV 35S promoter. Recombinant plasmids and *pCAMBIA1301* were transformed into *Agrobacterium* strain EHA105 and then introduced into Arabidopsis by *Agrobacterium*-mediated floral dip method [47]. The hygromycin-resistant positive transgenic individuals were screened from progeny seeds. T3 lines were obtained by screening generationally and used as the follow-up experimental materials after identification performed by PCR and qRT-PCR, whose identification primers were GUS-F/R and Q.GUS-F/R (Supplementary Table S1).

### 4.5. Histochemical GUS Staining

The seedlings and plant organs of each of the transgenic Arabidopsis lines were immersed in GUS staining solution (Solarbio LIFE SCIENCES, Beijing, China) for 4 h at 37 °C. Before staining, they were placed in a decompression chamber for 30 min. After staining, they were immersed in 70% alcohol for 3 h and then soaked in 95% alcohol until chlorophyll was completely removed at 37 °C. The rosette leaves of transgenic Arabidopsis treated with hormones were stained and decolorized in the same way. Some stained seedlings and leaves were observed and photographed with the Pixera Pro600ES digital CCD camera system.

### 4.6. The Analysis of GUS Activity

The analysis of GUS activity was performed as described in previous study [48]. The rosette leaves of transgenic Arabidopsis treated with phytohormones collected at different periods of time were ground in liquid nitrogen to extract samples to be tested. The 4-methyl-umbelliferyl-glucuronide (4-MUG) (Sangon Biotech, Shanghai, China) was used as a substrate. Fluorescence was determined using a multifunctional microplate reader (PerkinElmer, Shanghai, China) at excitation and emission wavelengths of 365 and 455 nm, respectively.

### 4.7. RNA Extraction and qRT-PCR Analysis 

The total RNA was isolated from columnar apple Wi seedlings with RNA Pure Plant Plus Kit (TIANGEN BIOTECH, Beijing, China). HiScriptII 1st strand cDNA synthesis kit (Vazyme, Nanjing, China) was used for synthesizing cDNA. qRT-PCR was performed with AceQ qPCR SYBR Green Master Mix (Vazyme, Nanjing, China). The *MdActin* gene, which was stably expressed in all of the tissue, was selected as a reference gene [13]. Relative expression levels were calculated using the comparative Ct method [48].

### 4.8. Statistical Analysis 

The data were shown as the means ± SE of three independent experiments with at least three replicates in each experiment. Differences among means were analyzed by one-way analysis of variance (ANOVA) combined with Duncan’s multiple range test: * p < 0.05, ** *p* < 0.01 and *** *p* < 0.001.

## 5. Conclusions

Multiple transcription factors involving TCP, BEL1 and BES/BZR1 and other transcription factor binding sites were predicted on the promoter of *MdCoL*. The *MdCoL* promoter was able to respond to ABA, BR and GA_3_, and these hormones could regulate the *MdCoL* expression. Moreover, GA and ABA could regulate the expression of *MdCoL* in a feedback regulatory loop to further affect the expression of columnar traits.

## Data Availability

The data presented in this study are available in the article and the Supplementary Materials.

## Supplementary Materials

The following supporting information can be downloaded at: https://www.mdpi.com/article/10.3390/ijms231810781/s1.
