# Supplementary material for: The Characterization of Columnar Apple Gene MdCoL Promoter and Its Response to Abscisic Acid, Brassinosteroid and Gibberellic Acid
Source: Int J Mol Sci. 2022 Sep 15;23(18):10781. doi: 10.3390/ijms231810781 (PMC9505010; doi:10.3390/ijms231810781)
Supplement: Supplementary file 1 [file ijms-23-10781-s001.zip › ijms-1867234-supplementary.pdf]

Supplementary Table S1. Primer Sequence.

Primer Name	Sequence (5′-3′)
MdCoLp1-F	GGATCCCCGTCATACTCGCCTTCTCG
MdCoLp2-F	GGATCCTTGGCCTACTCTCCTAATTGCTA
MdCoLp3-F	GGATCCCTCAACGGCCACACATGCTC
MdCoLp4-F	GGATCCGATTCGAACTTTGGCCGTAGAG
MdCoLp1-R	CCATGGATCAACCTTTGAAACACAAGAGTAAA
GUS-F	TAATGTTCTGCGACGCTCAC
GUS-R	ATCGGCGAAATTCCATACCT
Q.GUS-F	CGGTCAGTGGCAGTGAAGGG
Q.GUS-R	CGAGGTACGGTAGGAGTTGG
MdActin-F	ATGCCAGGGAACATGGTAGA
MdActin-R	TGAGCGAGAAATTGTCAGGG
