# Supplementary material for: The Characterization of Columnar Apple Gene MdCoL Promoter and Its Response to Abscisic Acid, Brassinosteroid and Gibberellic Acid
Source: Int J Mol Sci. 2022 Sep 15;23(18):10781. doi: 10.3390/ijms231810781 (PMC9505010; doi:10.3390/ijms231810781)
Supplement: Supplementary file 1 [file ijms-23-10781-s001.zip › ijms-1867234-SupplementaryMaterials.pdf]

Supplementary Materials

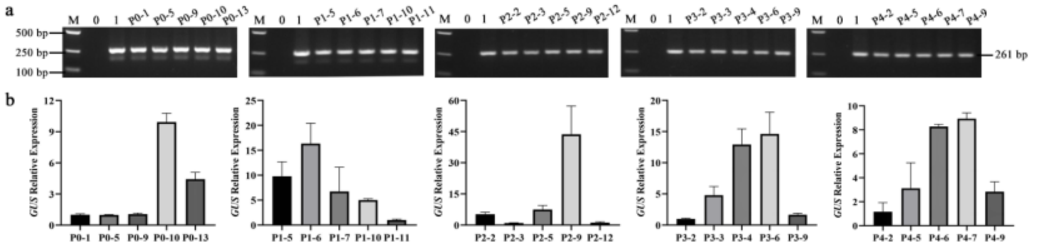

Figure S1. Identification of transgenic *Arabidopsis* plants. (a): Identification results of transgenic *Arabidopsis* in DNA level; (b): Identification results of transgenic *Arabidopsis* in RNA level. M: DL2000 Marker; 0: negative control; 1: positive control.
